# The time-dependent reproduction number for epidemics in heterogeneous populations

**DOI:** 10.1098/rsif.2025.0095

**Published:** 2025-07-09

**Authors:** Ioana Bouros, Robin N. Thompson, David J. Gavaghan, Ben Lambert

**Affiliations:** ^1^ Department of Computer Science, University of Oxford, Oxford, UK; ^2^ Mathematical Institute, University of Oxford, Oxford, UK; ^3^ Department of Statistics, University of Oxford, Oxford, UK

**Keywords:** epidemiology, renewal equation, heterogeneity, *R_t_* estimation

## Abstract

The time-dependent reproduction number $R_{t}$ can be used to track pathogen transmission and to assess the efficacy of interventions. This quantity can be estimated by fitting renewal equation models to time series of infectious disease case counts. These models almost invariably assume a homogeneous population. Individuals are assumed not to differ systematically in the rates at which they come into contact with others. It is also assumed that the typical time that elapses between one case and those it causes (known as the generation-time distribution) does not differ across groups. But contact patterns are known to widely differ by age and according to other demographic groupings, and infection risk and transmission rates have been shown to vary across groups for a range of directly transmitted diseases. Here, we derive from first principles a renewal equation framework which accounts for these differences in transmission across groups. We use a generalization of the classic M’Kendrick–von Foerster equation to handle populations structured into interacting groups. This system of partial differential equations allows us to derive a simple analytical expression for $R_{t}$, which involves only group-level contact patterns and infection risks. We show that the same expression emerges from both deterministic and stochastic discrete-time versions of the model and demonstrate through simulations that our $R_{t}$ expression governs the long-run fate of epidemics. Our renewal equation model provides a basis from which to account for more realistic, diverse populations in epidemiological models and opens the door to inferential approaches which use known group characteristics to estimate $R_{t}$.

## 1. Introduction

In the long run, an infectious disease epidemic grows if, on average, the number of new infections generated by each infectious individual exceeds one; contrastingly, it will subside if this value is below one. The (instantaneous) time-dependent reproduction number, $R_{t}$, is rigorously defined as the average number of secondary cases generated by an infected case at time t assuming that transmission conditions remain the same in the future. $R_{t}$ is an emergent property of a pathogen spreading through a specific population, and it depends on the biology of the pathogen and the characteristics of the population, including any measures taken to limit its spread [1,2]. Accordingly, determining $R_{t}$ is crucial for public health policymaking during epidemics.

Renewal equation models have, over the past two decades, become the predominant models used when $R_{t}$ is estimated from epidemiological time series, most commonly using time series of infectious disease case counts [3–5]. Their success is due to their relative simplicity: these models assume that new infections are caused by previous infections with time lags between parent and daughter infections; these time lags are assumed to be characterized by a *generation-time distribution* allowing for variation in this quantity across infector–infectee pairs [6]. In these models, the number of new infections caused by a typical new case at time t is given by $R_{t}$. Usually, these models are fitted to infectious disease case counts (rather than infection counts, which are harder to observe) and, in so doing, $R_{t}$ is estimated. There is a large literature and range of software tools devoted to $R_{t}$ inference through renewal equations [3–5,7–9].

These inferential frameworks are built upon an implicit assumption of uniformity in the population, i.e. that disease transmission rates are the same across the population. This ignores what is widely known—that disease transmission differs systematically across groups, and is explicitly modelled in other types of models of disease transmission dynamics. For example, compartmental models (e.g. [10]) are frequently structured by age, largely because, in many populations, the numbers and types of contact that individuals typically have with others depend strongly on their age. This is so widely recognized that there is a literature devoted to estimating so-called *contact matrices* that capture this information (e.g. [11]). Infection risk can also differ according to other demographics [12,13]. The time period between one case and those it causes can also vary according to demographics: pathogens may undergo distinct dynamics within individuals from specific groups and be spread differently, and members of different groups may also adapt their behaviour to varying degrees if symptoms show. These differences can result in large variations between generation times across groups (e.g. for COVID-19 [14]).

This shortcoming of the standard renewal framework has been recognized in previous work. It is possible to estimate a group-specific $R_{t}$ from infection time series by making assumptions about the rate at which pathogens spread within and between groups [15], although differences in generation-time distributions according to group have not been considered; in such frameworks, an overall reproduction number can be determined through a weighted average of within- and between-group reproduction numbers. An alternative approach is to model the population using infection-age-structured partial differential equation (PDE) systems, with one PDE for each of the groups [16]. This approach follows the classic M’Kendrick–von Foerster equation framework, which is central in the study of population dynamics in areas such as demography [17–19], where birth and death processes vary according to age and we follow this approach here.

We suppose there are groups distinct in terms of their contact behaviour, infection risks and generation-time distributions. Like [16], we determine an analytical expression for $R_{t}$, but ours is far simpler and does not involve the generation-time distribution. We also provide a rigorous derivation starting from an age-structured system of coupled PDEs, which makes clear the assumptions underpinning the renewal equation dynamics for a structured population, and crucially, this indicates special scenarios when the dynamics deviate from those shown in [16]. Using a discrete-time version of the model (as most inferential routines for $R_{t}$ use discrete-time renewal models), we arrive at the same expression for $R_{t}$. We use simulation to demonstrate that our expression for $R_{t}$ behaves as expected: $R_{t}=1$ defines the boundary between long-run epidemic growth (if $R_{t}>1$) and long-run decline (if $R_{t}<1$). We show that this $R_{t}$ expression holds also for a stochastic version of our model, where, on average, the behaviour of these systems is delineated by the $R_{t}=1$ boundary. We also derive a relationship between $R_{t}$ and the calendar time growth rate of the epidemic, $r_{t}$, for a population organized into groups, and we show that $r_{t}$ is particularly sensitive to changes in the generation time of groups with the most contacts. Our framework naturally allows known characteristics of different groups to be incorporated into renewal equation models of epidemics and into associated estimates of epidemiological quantities.

## 2. Methods

### 2.1. Renewal equations used to infer $R_{t}$

Renewal models are primarily used to estimate $R_{t}$, and we now describe the most basic, yet indicative, form of these models. This model is stochastic and discrete-time (typically with time-steps of one day) and assumes that the population is homogeneous and takes the form


(2.1) $$I_{t}∼\text{Poisson}(R_{t}\Lambda_{t})\text{, where }\Lambda_{t}=\sum_{a=1}^{t-1}w_{a}I_{t-a}.$$

In this expression, $I_{t}\geq 0$ represents counts of infections arising at time t; $\Lambda_{t}\geq 0$ is known as the *transmission potential* at time t, which is a weighted sum of past infections where the weights are determined by a *generation-time distribution*. This distribution is a discrete probability distribution, $\{w_{a}{\}}_{a=1}^{\infty }$, such that $\sum_{a=1}^{\infty }w_{a}=1$, where $w_{a}\geq 0$ gives the probability that the time elapsing between a past infection and a daughter case is a days.

### 2.2. Derivation of the renewal equation for the M’Kendrick–von Foerster model

Equation (2.1) is a stochastic renewal equation that can be motivated by the classic M’Kendrick–von Foerster model [17,18], which uses an age-structured PDE to model population dynamics where there are birth and death processes that depend on age (e.g. [19, chapter 1.7]). This model is deterministic and, typically when used in the context of epidemics, models the numbers of those infected continuously and is continuous in time and age of infection. We walk through a derivation of the renewal equation for this classic model since our approach for modelling structured populations extends it. In §2.3, we make explicit our assumptions in using this framework to model epidemic dynamics for a homogeneous population.

Following closely the derivation and notation used in [19, chapter 1.7], we denote the *density* of infections still present at time t which began a days ago by n(t,a); the total *number* of infections is then given by $\int_{0}^{\infty }n(t,a)\text{d}a$. Note that, in our version of this classic model, a denotes the infection age and *not* ages of infected individuals. In a small time increment dt, the conservation law for the population dictates that [19, chapter 1.7]


(2.2) $$\text{d}n(t,a)=\frac{\partial n}{\partial t}\text{d}t+\frac{\partial n}{\partial a}\text{d}a=\mu (a)\text{d}t,$$

where μ(a) is the rate at which infections end. In our work, we do not keep track of the number of recovered or dead cases, essentially assuming that μ(a)=0. Since infection age changes at the same rate as calendar time, dt/da=1, and equation (2.2) can then be simplified to the following PDE:


(2.3) $$\begin{array}{lrr} & \frac{\partial n}{\partial t}+\frac{\partial n}{\partial a}=0. & \end{array}$$

We specify a boundary condition which dictates the rate at which new infections arise:


(2.4) $$n(t,0)=\int_{0}^{\infty }b(t,a)n(t,a)\text{d}a,$$

where b(t,a)≥0,∀t,a is the rate at which infections arising a days ago generate new infections. In addition, we also assume that the birth rate of new infections arising from very old infections is effectively zero, that is for large positive values of a, b(t,a)=0,∀t. In what follows, we explicitly model only the population dynamics for t≥0, and, in order to close the system, we assume that at t=0, there is a density of infections given by


(2.5) $$\begin{array}{lcr} & n(0,a)=f(a). & \end{array}$$

We can consider two solution classes corresponding to distinct groups of infected individuals at time t, each of which has a different relationship between a and t:


(2.6) $$a=\left\{ \begin{array}{l} t+a_{0},\text{ for }a>t,\text{ i.e. for infections arising before }t=0,\\ t-t_{0},\text{ for }a\leq t,\text{ i.e. for infections arising from }t=0\text{ onwards}, \end{array}\right.$$

where $a_{0}>0$ is the age of an infection at time t=0 for the first group, and $t_{0}$ is the time at which an infection arises for the second (where $0\leq t_{0}\leq t$). For each of the cases in equation (2.6), we can write n(t,a)=n(t,a(t)):=n(t)*,* i.e. a function of t only, which we can substitute into equation (2.3) to yield an ordinary differential equation (ODE):


(2.7) $$\frac{\text{d}n}{\text{d}t}=0,$$

which is valid only along one of the so-called *characteristic* lines defined in equation (2.6). In this context, the characteristics correspond to infections arising at a particular point in time, τ: if a>t, a unique value of $a_{0}$ gives τ<0; if a≤t, $\tau =t_{0}$. For each infection onset time then equation (2.7) has the solution


(2.8) $$\begin{array}{lcr} & n(t,a(t))=\text{constant}, & \end{array}$$

meaning that the number of individuals infected at a given time t remains forever constant: this makes sense because, in our framework, we do not allow for cessation of an infection.

We now derive these constant population sizes for each of the cases in equation (2.6) by considering these at conveniently chosen moments. When a>t, we can write $a=t+a_{0}$ meaning $n(t,t+a_{0})=\text{constant}$*,* and when t=0, the population size is


(2.9) $$\begin{array}{lcr} & n(0,a_{0})=f(a_{0})=f(a-t). & \end{array}$$

When a≤t, we can write $a=t-t_{0}$ and $n(t,t-t_{0})=\text{constant}$, and, when $t=t_{0}$:


(2.10) $$\begin{array}{lcr} & n(t_{0},0)=n(t-a,0). & \end{array}$$

Collecting equations (2.9) and (2.10), we have the solutions for the population sizes for all infection onset times:


(2.11) $$\begin{array}{lcr} & n(t,a)=\left\{ \begin{array}{c} f(a-t),a>t,\\ n(t-a,0),a\leq t. \end{array}\right. & \end{array}$$

Substituting equation (2.11) into equation (2.4) results in


(2.12) $$n(t,0)=\underset{a\leq t}{\underbrace{\int_{0}^{t}b(t,a)n(t-a,0)\text{d}a}}+\underset{a>t}{\underbrace{\int_{t}^{\infty }b(t,a)f(a-t)\text{d}a}}.$$

To avoid having to specify the initial conditions at t=0, we generally assume that t is large such that $b(t,a^{\prime })\approx 0,\forall a^{\prime }>t$. This assumption does not impact the early outbreak dynamics, as it simply determines that all active infections before the start time of the epidemic are ‘reclocked’ to have occurred at time zero with this equation (2.12) reduces to


(2.13) $$n(t,0)=\int_{0}^{t}b(t,a)n(t-a,0)\text{d}a.$$

Equation (2.13) gives a recurrence relation for our system solution, which is an implicit equation for n(t,0). In §2.3, we show how this can be explicitly solved.

We now define I(t):=n(t,0) to represent a measure of new infections arising at time t, and equation (2.13) then becomes


(2.14) $$I(t)=\int_{0}^{t}b(t,a)I(t-a)\text{d}a.$$

Equation (2.14) is a continuous and deterministic renewal equation.

### 2.3. The M’Kendrick–von Foerster model of an epidemic in a homogeneous population

We now make explicit our assumptions around how infections arise in a homogeneous population and adapt equation (2.14) accordingly. We then derive long-term solutions for this model. We suppose that there are two distinct time-dependent processes that modulate the birth rate of new infections: (i) the total number of individuals an infected person comes into contact with over the course of their infectious period if their infection started at time t, C(t)≥0; and (ii) the probability, 0≤γ(t)≤1, that each contact results in an infection; this implicitly assumes that individuals do not change their behaviour as their infection progresses; for example, it assumes that when symptoms appear infected individuals do not change how they socialize. We assume that there is also an infection-age-dependent process which governs how much time typically elapses between one infection starting and those it causes: we use a continuous probability distribution for this, with a probability density function, w(a). The birth rate of new infections is then given by: b(t,a)=C(t)γ(t)w(a)*,* and we can substitute this into equation (2.4) to produce a renewal equation for new infections:


(2.15) $$I(t)=\int_{0}^{t}C(t)\gamma (t)w(a)I(t-a)\text{d}a.$$

The rate at which new infections are generated from past infections is, in this model, not explicitly contingent on the numbers of those infected in the past. This differs from, say, an SIR compartmental model, where the rate at which new infections are generated from past infections depends on the availability of susceptible individuals, which diminishes as the epidemic spreads through a population. In addition, we do not consider the termination of an infection; that is the death rate of new infections is assumed to be μ(a)=0.

If we consider time-constant C=C(t) and γ=γ(t), the function I(t) is a convolution of past values of itself with a time-invariant kernel w(a). The only function which has this property is an exponential; this means that I(t) can only grow or decline exponentially over time. An alternative way to see that only unregulated growth or decline is allowed is because equation (2.15) lacks self-regulation, for example, due to the removal of susceptible individuals. This means that the system has no non-zero infection equilibrium.

We then look for a similarity solution [19, chapter 1.7] of our system (for large t) of the form


(2.16) $$\begin{array}{lcr} & n(t,a)=\exp(rt)p(a), & \end{array}$$

where r is the growth rate of the epidemic (which can be negative if there is epidemic decline), and p(a) is an age-dependent distribution. Substituting equation (2.16) into the a≤t solution in equation (2.11) which has n(t,a)=n(t−a,0) gives


(2.17) $$\begin{array}{lcr} & p(a)=p(0)\;\exp(-ra), & \end{array}$$

which we substitute back into equation (2.16) to give us the solution:


(2.18) $$\begin{array}{lcr} & n(t,a)=p(0)\;\exp(r(t-a)). & \end{array}$$

Consequently, the number of new infections grows (or declines) exponentially over time:


(2.19) $$\begin{array}{lcr} & I(t)=p(0)\;\exp(rt), & \end{array}$$

as desired for our intended solution. Substituting this into equation (2.15) and dividing both sides by common terms gives


(2.20) $$1=\int_{0}^{t}C(t)\gamma (t)w(a)e^{-ra}\text{d}a,$$

which is known as the Euler–Lotka equation and was first derived by Lotka at the start of the 20th century; this extended a more specific result obtained by Euler in the 1700s [20].

Whether the infected population grows or declines exponentially and the rate at which it does so is determined by the value of r which solves equation (2.20). There are no simple closed-form solutions to equation (2.20); instead, we show how the sign of r is determined by particular terms in this equation.

**Figure 1.**
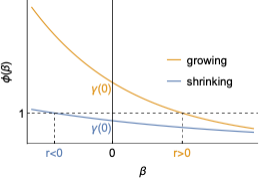
How the growth rate of an epidemic is determined by *ϕ(0).* Here, *$\phi (\beta ):=\int_{0}^{t}C(t)\gamma (t)w(a)e^{-\beta a}da$.* This figure is a reproduction of a figure from [19, chapter 1.7].

We define the function $\phi (\beta ):=\int_{0}^{t}C(t)\gamma (t)w(a){\text{e}}^{-\beta a}\text{d}a$; we note that, using this notation, r:={β, s.t. ϕ(β)=1}. Crucially, this function is monotonically decreasing in β. This means that if ϕ(0)>1, then r>0; if ϕ(0)=1, r=0; and if ϕ(0)<1, r<0 (see figure 1).

The sign of r is so determined by the value of ϕ(0):


(2.21)ϕ(0)=∫0tC(t)γ(t)w(a)da,(2.22)=C(t)γ(t)∫0tw(a)da,(2.23)≈C(t)γ(t), for large t.

Specifically, the population grows if C(t)γ(t)>1, is maintained if C(t)γ(t)=1 and shrinks if C(t)γ(t)<1. This behaviour is exactly the type of thresholding behaviour that is expected from a reproduction number, so we define


(2.24) $$\begin{array}{lcr} & R(t):=C(t)\gamma (t). & \end{array}$$

This time-varying reproduction number acts intuitively: C(t)γ(t) is a measure of infections generated over the course of an infection (if transmission remains the same). If it exceeds 1, then each infection more than replaces itself; and analogously for the other two cases.

Formally, the reproduction number is defined as the largest (and here only) eigenvalue of the *next-generation operator* [21]; this is here an integral operator, K:


(2.25) $$K(g(a)):=\int_{0}^{t}C(t)\gamma (t)w(a)g(a)\text{d}a.$$

If g(a)=1, equation (2.25) yields the expected number of infections generated by an infection, i.e.


(2.26) $$\begin{array}{lcr} & K(1)=C(t)\gamma (t), & \end{array}$$

meaning R(t)=C(t)γ(t) as required. In practice, R(t) is written as $R_{t}$.

### 2.4. Reproducing our simulation results

Throughout this paper, we use simulations of unfolding outbreaks to support our mathematical derivations. To allow others to rerun these simulations, we make our Python code available through a public GitHub repository [22].

## 3. Results

In our results, we use a mix of mathematically derived logic complemented with simulations to back up our theory. Our simulations aim to demonstrate support for our derivations rather than closely matching real outbreak scenarios.

### 3.1. A renewal equation for new infections in a structured M’Kendrick–von Foerster model

We now extend the model presented in §2 and assume that the population is structured into interacting groups. In this section, we derive the renewal equation for new infections in this model.

We assume that the groups within the population differ in two ways that affect disease transmission.

#### 3.1.1. Differences in contact patterns

We take inspiration from compartmental ODE models where contact patterns within and between groups are incorporated through contact matrices (e.g. [11]).

#### 3.1.2. Differences in within-host pathogen dynamics and behaviour during infection

Within-person pathogen dynamics and transmission patterns can differ across individuals. Individuals can also differ in terms of their behaviour throughout infection: when symptoms show, more risk-averse individuals may choose to avoid socializing to prevent infecting others. Each of these characteristics could differ systematically across, for example, demographic groups, leading to differences in the generation time across these groups.

We define $n^{i}(t,a)$ as the density of current infections in population group i∈{1,2,...,N} present at time t that arose a time periods ago. We can stack these densities into a vector containing this information for all groups:


$$\begin{array}{r} \underline{n}(t,a)=\left(\begin{array}{c} n^{1}(t,a)\\ \vdots \\ n^{N}(t,a) \end{array}\right). \end{array}\;$$

As for the homogeneous population model, applying population conservation yields the following system of PDEs:


(3.1) $$\frac{\partial n}{\partial t}+\frac{\partial n}{\partial a}=0,$$

where $\partial \underline{n}/\partial x:=[\partial n^{1}/\partial x,\partial n^{2}/\partial x,...,\partial n^{N}/\partial x{]}^{\prime }$ is the vector-partial derivative. We denote the birth rate of infections in group i which were caused by infections in group j by $b^{j\to i}(t,a)$. This means that the total density of new infections in group i at time t is given by those caused by infections across all groups:


(3.2) $$n^{i}(t,0)=\sum_{j=1}^{N}\int_{0}^{\infty }b^{j\to i}(a)n^{j}(t,a)\text{d}a.$$

As for the homogeneous model (§2.3), we assume that the birth rate of new infections is the product of three terms:


(3.3) $$\begin{array}{lcr} & b^{j\to i}(t,a)=\gamma (t)w^{j}(a)C^{\left(ji\right)}(t), & \end{array}$$

where $C^{\left(ji\right)}(t)$ now represents an element from a contact matrix and indicates the total number of contacts in group i made by an individual in group j over the course of their infection; $w^{j}(a)$ indicates a probability density function representing the generation time of group j. We make the additional assumption that the probability that each contact results in an infection γ(t) is the same over all combinations of population groups. An alternative way to view this assumption is when setting γ(t)=1 meaning $C^{\left(ji\right)}(t)$ represents the total number of infections caused by group i in those of category j throughout their infection—this implicitly assumes that the contact matrix accounts for variations in infectiousness and susceptibility across infector/infectee groups.

We can then use equations (3.2) and (3.3) to write an expression giving the rate of new infection generation across all groups:


n_(t,0)=(∑j=1N∫0∞γ(t)wj(a)C(1j)(t)nj(t,a)da⋮∑j=1N∫0∞γ(t)wj(a)C(Nj)(t)nj(t,a)da)=∫0∞γ(t)(∑j=1NC(1j)(t)wj(a)nj(t,a)⋮∑j=1NC(Nj)(t)wj(a)nj(t,a))da=∫0∞γ(t)C(t)(w1(a)n1(t,a)⋮wN(a)nN(t,a))da=∫0∞γ(t)C(t)[w1(a)⋱wN(a)]⏟W(a)(n1(t,a)⋮nN(t,a))da(3.4)=∫0∞γ(t)C(t)W(a)(n1(t,a)⋮nN(t,a))da.

As $\int_{0}^{\infty }w^{i}(a)da=1,\forall i$*,* the matrix W(a) satisfies the following property:


(3.5) $$\int_{0}^{\infty }W(a)\text{d}a=I_{N}.$$

In order to close the system, we assume that at t=0, there is a density of infections given by


(3.6) $$\underline{n}(0,a)=\underline{f}(a).$$

We use the same approach as for the homogeneous population model to arrive at the analogous form of equation (2.12) for the structured population model:


n_(t,0)=∫0∞γ(t)C(t)W(a)n_(t,a)da(3.7)=∫0tγ(t)C(t)W(a)n_(t−a,0)da+∫t∞γ(t)C(t)W(a)f_(t−a)da.

As before, we assume that t is large such that infections with age exceeding this time contribute negligibly to onward transmission, and equation (3.7) then reduces to


(3.8) $$\underline{n}(t,0)=\int_{0}^{t}\gamma (t)C(t)W(a)\underline{n}(t-a,0)\text{d}a,$$

which can be written as a renewal equation for new infections:


(3.9) $$\underline{I}(t)=\int_{0}^{t}\gamma (t)C(t)W(a)\underline{I}(t-a)\text{d}a.$$

### 3.2. The time-varying reproduction number for structured populations

The multiple-group renewal equation, equation (3.9), appears of the same form as the equivalent expression for a single group, equation (2.14). It is natural then to suppose that the long-term solution for the multiple-group model should be of analogous form:


(3.10) $$\underline{I}(t)={\text{e}}^{rt}\underline{\Phi },$$

where $\underline{\Phi }$ is a constant non-negative vector.

In appendix A, we outline the set of conditions for equation (3.10) to hold. We then prove that these conditions hold in general for realistic population structures where groups interact; the only exceptions are populations in which not all population groups mix (as seen in equation (A 1)) and diseases where the generation-time interval w has only one non-zero entry (that is subsequent infections occur exactly j days after the parent infection for w(j)≠0).

In the case of non-interacting populations, for example, it is not generally true that the growth rate of new infections should be identical across groups. For example, if groups have differing generation times and they do not interact, the infections will generally grow at different calendar rates in each group. This type of situation could arise if intervention policies were put in place which completely isolated parts of the population; our theory requires only that the contact rate between these parts of the population and others is non-zero, however, and it is unlikely that such a policy would entirely isolate these groups.

We demonstrate that equation (3.10) holds using simulations of a discrete deterministic renewal model; we set parameters of the model such that there is epidemic growth. In these simulations, we assume that the population is structured into three age groups: 0- to 20-year-olds, 20- to 65-year-olds and those aged 65+. We use a contact matrix derived from [11] as proxy for the total contact matrix for this population:


(3.11) $$\begin{array}{lcr} & \begin{array}{r} C(t)=\left(\begin{array}{ccc} 6.33 & 1.61 & 0.1\\ 4.65 & 7.41 & 0.51\\ 0.49 & 1.17 & 1.13 \end{array}\right), \end{array} & \end{array}$$

where the rows correspond to infectors aged 0−20 (first row), 20−65 (second row) and 65+ (third row); the columns correspond to infectees in groups with the corresponding ordering as for the rows. The values in the matrix represent the average daily contacts an individual in the row age group has with those in the column age group. Those aged 0−20 have the vast majority of their contacts with members of the same group; those aged 20−65 have high contact rates with the two youngest age groups; those aged 65+ have comparably low contact rates across all age groups.

In table 1, we summarize different generation interval scenarios that we will use in our following analyses. All generation intervals presented follow a Gamma distribution, with stated means and standard deviation. While the values we utilized in our analyses may not be taken from a clinical study, they mean to demonstrate theoretically the importance of a correct assessment of R(t), when not all population groups follow identical generation times.

**Table 1.** Generation-time distributions across age groups used in our simulation scenarios. Scenario 7 is only used in producing electronic supplementary material, figure S1 in appendix A.3.

		mean generation time			standard deviation generation time		
scenario	description	0−20	20−65	65+	0−20	20−65	65+
1	mixture of long and short-generation times, varied across groups	5.6	15.3	25	3.3	3.3	3.3
2	medium length generation times, equal across groups	15.3	15.3	15.3	3.3	3.3	3.3
3	mixture of long and short-generation times, swapped and varied across groups	25	15.3	5.6	3.3	3.3	3.3
4	short-generation times, varied across groups	3.3	5	7	3.3	3.3	3.3
5	short-generation times, equal across groups	5.1	5.1	5.1	3.3	3.3	3.3
6	mixture of long- and short-generation times, varied across groups with different standard deviations	15.3	3.5	7	5.3	2.5	3
7	long-generation times, varied across groups	30	25.3	15	3.3	3.3	3.3

**Figure 2.**
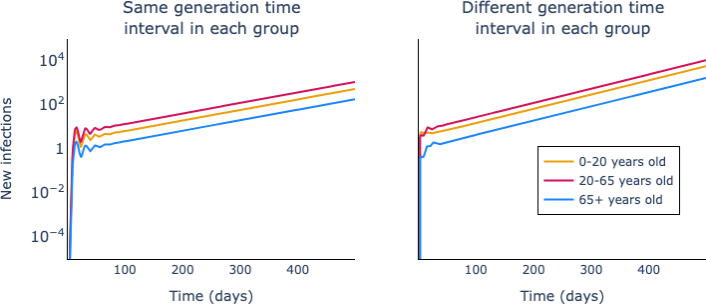
Epidemic growth occurs at the same rate across interacting groups. For both panels, we use the same reproduction number *R(t)=1.2* and simulate using a discrete deterministic renewal equation. In the left-hand panel, the generation time distributions are given by scenario 2 in table 1; in the right-hand panel, we assume the generation-time distributions differ across groups and are given by scenario 1 of the same table. The contact matrix assumed is given in equation (3.11)*.*

In figure 2, we show the results of these simulations; the right-hand panel shows the evolution of the outbreaks in each group when the groups all have the same generation-time distribution; the left-hand panel shows the equivalent when the generation-time distributions differ across groups. In both cases, the rate of outbreak growth is the same across the three populations, as is predicted by equation (3.10).

Because equation (3.10) holds for realistic populations, we assume a solution of this form and substitute it into equation (3.9). Simplifying both sides, the resulting equation reads as


(3.12) $$\underline{\Phi }=(\int_{0}^{\infty }\gamma (t)C(t)W(a){\text{e}}^{-ra}\text{d}a)\underline{\Phi }:=\bar{K}(r)\underline{\Phi },$$

where $\bar{K}(\beta ):=\left(\int_{0}^{\infty }\gamma (t)C(t)W(a){\text{e}}^{-\beta a}\text{d}a\right)$ is the Laplace integral transform of the positive matrix $\gamma (t)C_{t}W(a)$. Equation (3.12) implies that the vector $\underline{\Phi }$ is an eigenvector of the positive matrix $\int_{0}^{\infty }\gamma (t)C(t)W(a){\text{e}}^{-ra}\text{d}a$, with associated eigenvalue 1.

We now use a linear algebra result called the *Perron–Frobenius* theorem [23] to convert equation (3.12) into a form to which we can apply the same logic as for the single-population model in §2.3. This theorem states that for a positive matrix, in our case K(β), the following three results hold:

(1) defining the eigenvalues of $\bar{K}(\beta )$ as $\nu_{1},\nu_{2},...,\nu_{N}$*,* the spectral radius ρ(K(β)):=max⁡({|ν1|,|ν2|,...,|νN|})*,* is an eigenvalue of K(β);
(2) ρ(K(β))∈ℝ and ρ(K(β))>0;
(3) the eigenvector $\underline{v}$ of K(β) associated to the spectral radius $\rho (\bar{K}(\beta )$ is a positive vector and the one and only non-negative eigenvector of K(β) (all other non-negative eigenvectors are multiples of $\underline{v}$).

All values of the vector $\underline{I}(t)$ are non-negative meaning that $\underline{\Phi }$ must also be a non-negative vector. Additionally, $\underline{\Phi }$ is an eigenvector of K(r), which corresponds to an eigenvalue of 1 and thus satisfies condition 2. Then condition 3 in the Perron–Frobenius theorem indicates that the spectral radius of K(r) is equal to 1, i.e.


(3.13) 1=ρ(K(r)).

Equation (3.13) is of a similar form to equation (2.20) for the one-group model. Like the right-hand side of equation (2.20), the function ρ(K(r)), the spectral radius of the Laplace integral transform of a positive matrix, is also monotonically decreasing in r [24]—this means that we can apply the same reasoning as for the single-group model.

Since K(0) is the next-generation operator [21] yielding the expected numbers of newly infected in each of the groups assuming a single infected individual in each group, we define the overall reproduction number as


R(t)=ρ(K¯(0))=ρ(∫0∞γ(t)C(t)W(a)e0ada)=ρ(γ(t)C(t)∫0∞W(a)da)=ρ(γ(t)C(t))(3.14)=γ(t)ρ(C(t)).

Since the spectral radius of the Laplace integral transform K(r) of a positive matrix is monotonically decreasing [24], we find that


(3.15) Epidemic growth⟺r>0⟺ρ(K(0))>ρ(K(r))⟺R(t)=γ(t)ρ(C(t))>1,

and similarly so with R(t)<1 indicating long-term outbreak declines. We then define the time-varying reproduction number for a structured population, R(t), by


$$\begin{array}{c} R(t)=\gamma (t)\rho (C(t)). \end{array}$$

As discussed in §2, renewal equations when used in practice are usually discrete; in appendix B, we show that the same expression for $R_{t}$ arises from the analogous discrete renewal model for a structured population. We use this discrete renewal model in the majority of our simulations, which is given by


(3.16) $$\begin{array}{lrlr} & I_{t}^{\left(j\right)} & =\frac{R_{t}}{\rho (C_{t})}\sum_{i=1}^{N}C_{t}^{\left(ji\right)}\sum_{a=1}^{t-1}w_{a}^{\left(i\right)}I_{t-a}^{\left(i\right)}, & \end{array}$$

where $C_{t}^{\left(ji\right)}$ represents the (i,j)th element of a matrix of total contacts made over the course of an infection started at time t^1^; $\rho (C_{t})$ is the largest eigenvalue of this contact matrix; $\left\{w_{a}^{\left(i\right)}\right\}$ represent elements of a discrete generation-time interval distribution for group i.

### 3.3. The long-run epidemic fate does not depend on the generation-time interval

Equation (3.14) indicates that the reproduction number does not depend on the generation times of individual groups, which is a generalization of results from previous work [16]. This makes intuitive sense because, in the long-run, an epidemic grows only if each infected case on average causes more than one subsequent infection—it does not depend on *when* those infections occur, which is what the generation-time encodes.

We now use simulations from equation (3.16) to illustrate this. We again assume that the population is structured into three age groups as described in §3.2.

**Figure 3.**
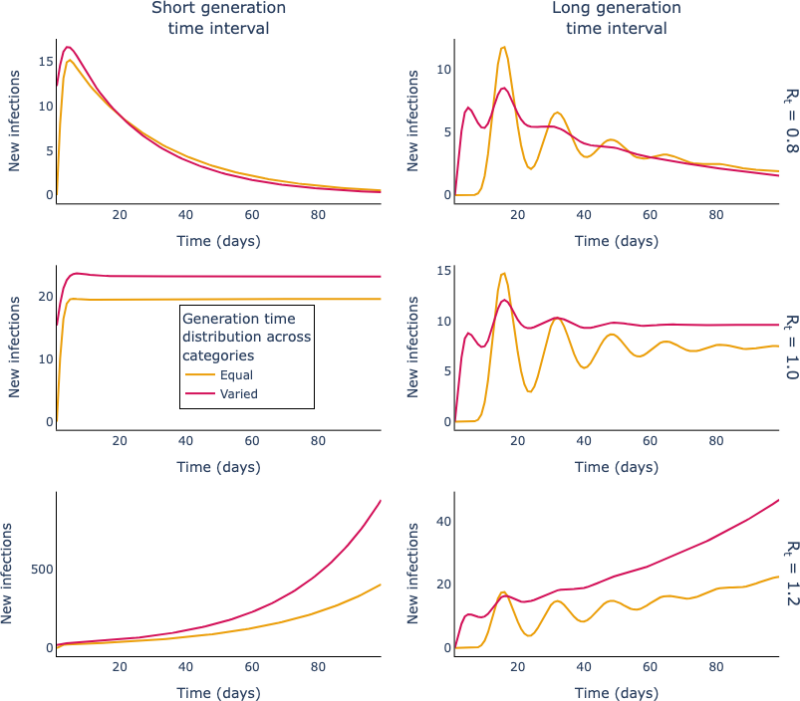
Whether an outbreak shrinks, stabilizes or grows in the long-term does not depend on the generation-time distribution. The rows correspond to different *$R_{t}$* values, increasing from top to bottom. The columns correspond to short (left) and longer (right) generation times. The yellow lines show aggregate outbreak dynamics (i.e. the evolution of the sum of new infection counts across the three groups) when the generation-time distribution is the same across all groups (given by scenarios 5 and 2 in table 1 for the short and long-generation-time intervals, respectively); the red lines show the equivalent quantity when there is variation in the generation-time distributions across the groups (the assumed generation times correspond to scenario 4 for the short-generation-time intervals and scenario 1 for the long-generation-time intervals in table 1).

In figure 3, we show the results of our simulations of unfolding outbreaks. This first set of simulations is based on two assumptions around variation in the generation-time distribution across the three groups: (i) when the three groups have the same generation-time distribution (red lines), or (ii) each of the groups have different generation time distributions (yellow lines). We explore how the mean of the generation-time distribution affects outbreak trajectories: the left-hand column shows simulations when the mean generation-time interval is typically short; the right-hand column shows simulations when the generation time interval is generally longer. Descending the rows corresponds to increasing $R_{t}$ values.

Figure 3 shows that the $R_{t}$ threshold determines whether, ultimately, an outbreak shrinks (top row: $R_{t}=0.8$), stabilizes (middle row: $R_{t}=1$) or grows (bottom row: $R_{t}=1.2$). The assumed form of the generation-time distribution across the groups does not affect whether longer term infection counts decline, stabilize or grow. However, changes to the generation-time distribution do affect the rate in calendar time at which infection counts change in the long term, and this is supported by further simulations (see appendix C).

For short-generation-time intervals, the infection counts reach their smooth long-term trajectories rapidly after an initial period where they respond to the initial conditions. For long generation times, particularly when the generation-time distribution varies by group, there are substantial oscillations in the infection counts, which appear to lessen with time.

These oscillations arise at the beginning of the simulations, when the system responds to the initial conditions before reaching the long-run growth trajectories. They are more marked when the generation time is long because this effectively introduces a lag between one clump of infections and those infections cause.

### 3.4. Stochastic models have the same $R_{t}$ threshold governing their mean behaviour

We now consider stochastic renewal models. In appendix D, we show that the reproduction number in a stochastic, discrete, single-population renewal model governs the long-term fate of the mean of the stochastic process; this proof carries directly over to the multiple-group case. We now illustrate through simulation that the expression derived for $R_{t}$ for the continuous model case (equation (3.14)) governs the behaviour of the mean of a stochastic renewal process of the form


$$\begin{array}{rl} I_{t}^{\left(j\right)} & ∼\text{Poisson}(\frac{R_{t}}{\rho (C_{t})}\sum_{i=1}^{N}C_{t}^{\left(ji\right)}\sum_{a=1}^{t-1}w_{a}^{\left(i\right)}I_{t-a}^{\left(i\right)}), \end{array}$$

where $I_{t}^{\left(j\right)}$ denotes the incidence in group j. The dynamics of the aggregate incidence across all groups is then given by


(3.17) $$\begin{array}{lrr} & I_{t}∼\text{Poisson}(\frac{R_{t}}{\rho (C_{t})}\sum_{j=1}^{N}\sum_{i=1}^{N}C_{t}^{\left(ji\right)}\sum_{a=1}^{t-1}w_{a}^{\left(i\right)}I_{t-a}^{\left(i\right)}). & \end{array}$$

We perform simulations of equation (3.17) and its deterministic counterpart in a three-group population assuming a contact matrix of the form given by equation (3.11).

**Figure 4.**
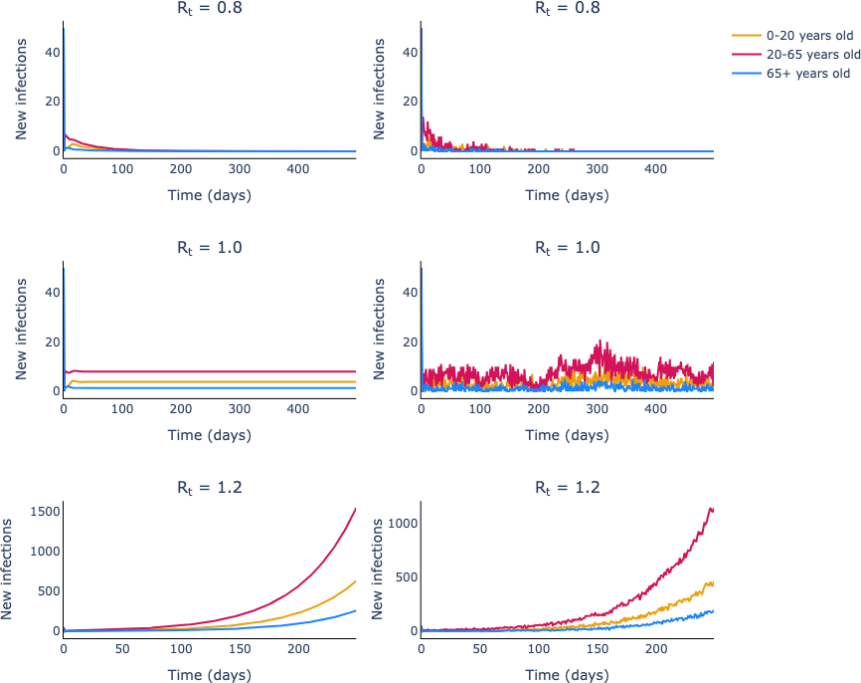
The long-term qualitative behaviour of incidence is determined in stochastic multiple-group renewal models by the same *$R_{t}$* expression as for deterministic models. The left-hand column shows simulations from a deterministic model and the right-hand column shows simulations from the stochastic model. The rows dictate the *$R_{t}$* values assumed. For all the populations modelled, we used the same age-specific generation-time intervals as given by scenario 6 in table 1.

In figure 4, we show that $R_{t}=\gamma_{t}\rho (C_{t})=1$ is the boundary that dictates the fate of the aggregate incidence for the deterministic model (left-hand column) and, analogously, for the long-run mean aggregate incidence of the stochastic process.

### 3.5. The relationship between $R_{t}$ and growth rate r for structured populations

Considering a homogeneous population, if equation (2.20) is divided through by R(t)=C(t)γ(t), we obtain


(3.18) $$\frac{1}{R(t)}=\int_{0}^{t}w(a)\exp(-ra)\text{d}a,$$

which previous studies have shown represents the relationship between R(t), the growth rate per infected individual and r, the growth rate of the epidemic size in calendar time [25]. We now derive an analogous relationship for a structured population. To do so, we take equation (3.13) and divide this through by R(t)=γ(t)ρ(C(t)):


(3.19) $$\frac{1}{R(t)}=\frac{\rho (\bar{K}(r))}{\gamma (t)\rho (C(t))}=\frac{\gamma (t)\rho (C(t)\int_{0}^{\infty }W(a)\exp(-ra)\text{d}a)}{\gamma (t)\rho (C(t))}=\frac{\rho (C(t)\int_{0}^{\infty }W(a)\exp(-ra)\text{d}a)}{\rho (C(t))}.$$

This shows that the relationship between R(t) and r depends on the contact patterns in the population and the individual group generation-time distributions. Interestingly, it does not depend on γ(t), the probability that a contact results in an infection (since we have modelled this as not varying by group).

An analogous relationship holds between R(t) and the doubling time of the epidemic (see appendix E).

We now demonstrate that the relationship between $R_{t}$ and r holds using simulations of an equivalent discrete-time model, which has a corresponding relationship between these two quantities:


(3.20) $$\begin{array}{lcr} & \frac{1}{R_{t}}=\frac{\rho (C_{t}\sum_{a=0}^{\infty }W_{a}(1+r{)}^{a})}{\rho (C_{t})}, & \end{array}$$

where $W_{a}$ is the diagonal matrix of the discretized generation-time intervals evaluated at the discrete-time interval a.

**Figure 5.**
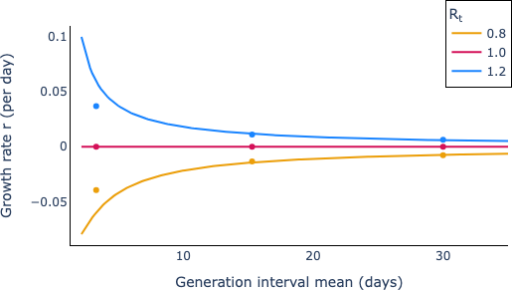
Theoretical and simulation-based relationships between epidemic growth and generation-time means largely agree. We represent in different colours the three possible epidemic long-term behaviours: epidemic growth (*$R_{t}=1.2$,* blue), epidemic persistence (*$R_{t}=1$*, red) and decay (*$R_{t}=0.8$*, yellow). The solid lines indicate the theoretical trajectories given by equation (3.20) (computed as seen in appendix G, and the points represent analogous estimates derived from simulations of the stochastic model.

In figure 5, we show the relationship between r and the mean of the generation time across three values of $R_{t}$. The lines show the theoretical quantities predicted by equation (3.20), which were determined using the SciPy optimizer method (see appendix G); the points show the empirical results derived from simulations of the stochastic renewal equation as given by equation (3.17). This plot shows that the theoretical and empirical results are in reasonable agreement. It also shows that as the generation-time mean increases, the rate of change of epidemic size in calendar time declines (for those cases when $R_{t}\neq 1$).

#### 3.5.1. Same generation-time distributions across groups

If the generation-time distribution across all groups is the same, we can write $W(a)=w(a)I_{N}$ and then use this to simplify equation (3.19):


$$\begin{array}{rl} \frac{1}{R(t)} & =\frac{\rho (C(t)\int_{0}^{\infty }w(a)I_{N}{\text{e}}^{-ra}\text{d}a)}{\rho (C(t))}=\frac{\rho (C(t)(\int_{0}^{\infty }w(a){\text{e}}^{-ra}\text{d}a))}{\rho (C(t))}\\ & =\frac{(\int_{0}^{\infty }w(a){\text{e}}^{-ra}\text{d}a)\rho (C(t))}{\rho (C(t))}=\int_{0}^{\infty }w(a){\text{e}}^{-ra}\text{d}a, \end{array}$$

to recover equation (3.18). So, when all groups share the same generation-time distribution, contact patterns do not affect the relationship between R(t) and r.

#### 3.5.2. Homogeneous mixing

We now suppose that all groups intermix at the same rate and derive a relationship between R(t) and r in this limit. Assuming groups mix homogeneously amounts to assuming an N×N contact matrix of the form


$$C(t)=c\left[\begin{array}{ccc} 1 & \ldots & 1\\ \vdots \; & \ddots & \vdots \;\\ 1 & \ldots & 1 \end{array}\right].$$

In this case, ρ(C(t))=cN (see appendix H) meaning the reproduction number R(t)=cNγ(t). We now consider:


$$C(t)\int_{0}^{\infty }W(a){\text{e}}^{-ra}\text{d}a=\left[\begin{array}{ccc} c\int_{0}^{\infty }w^{1}(a){\text{e}}^{-ra}\text{d}a & \ldots & c\int_{0}^{\infty }w^{N}(a){\text{e}}^{-ra}\text{d}a\\ \vdots & \ddots & \vdots \\ c\int_{0}^{\infty }w^{1}(a){\text{e}}^{-ra}\text{d}a & \ldots & c\int_{0}^{\infty }w^{N}(a){\text{e}}^{-ra}\text{d}a \end{array}\right],$$

which has spectral radius $\rho (C(t)\int_{0}^{\infty }W(a){\text{e}}^{-ra}\text{d}a)=c\int_{0}^{\infty }\sum_{i=1}^{N}w^{i}(a){\text{e}}^{-ra}\text{d}a$ (see Appendix H). This suggests the following relationship between the reproduction number and growth rate:


$$\begin{array}{r} \frac{1}{R(t)}=\frac{c\int_{0}^{\infty }\sum_{i=1}^{N}w_{s}^{i}{\text{e}}^{-ra}\text{d}a}{cN}=\int_{0}^{\infty }(\frac{1}{N}\sum_{i=1}^{N}w^{i}(a)){\text{e}}^{-ra}\text{d}a, \end{array}$$

which is equivalent to that of a single population with a generation-time distribution with elements $\bar{w}(a):=\frac{1}{N}\sum_{i=1}^{N}w^{i}(a)$; in other words, with a generation-time distribution equal to the average across all groups.

## 4. Discussion

The time-dependent reproduction number, $R_{t}$, is a critical measure for tracking pathogen transmission during an infectious disease epidemic. Modelling frameworks for inferring $R_{t}$ have tended to neglect heterogeneities between host groups in the population under consideration. However, heterogeneous mixing patterns and differences in the susceptibility and progression of a disease across groups can play an important role in shaping the epidemic trajectory. Here, we have developed a renewal equation framework enabling $R_{t}$ to be determined while accounting for these heterogeneities.

Our analyses produced an expression for $R_{t}$ which, as expected, is not contingent on the generation-time distribution; we show that our $R_{t}$ expression determines the long-term qualitative dynamics of the epidemic (i.e. whether case numbers eventually grow or decline) if transmission conditions remain the same. This is despite the fact that the generation-time sets the relationship between $R_{t}$ and the epidemic growth rate (figure 5). Finally, we showed that our $R_{t}$ expression also governs the expected long-term dynamics of stochastic renewal equation models.

For the stochastic renewal equation underlying parts of our analysis, we assumed that the number of cases each day is drawn from a Poisson distribution. However, more over-dispersed distributions can be used in renewal equation models, including accounting for the possibility of super-spreading events [26,27]. If we replace the Poisson distribution with an overdispersed cousin with the same mean, our theory (see appendix D) indicates that the same threshold conditions apply.

The research presented here builds on a substantial amount of literature on the topic of $R_{t}$ inference. Initial methods for estimating $R_{t}$ have been extended in multiple directions, including allowing for different strains of a pathogen [28] and inference of the generation time or serial interval distribution [5]. In recent work, temporal aggregation of disease incidence data (e.g. weekly rather than daily case reporting) has been accounted for in $R_{t}$ inference methods [29,30]. Despite these extensions, an exhaustive analysis of $R_{t}$ quantification in populations consisting of multiple host groups has not been undertaken until now.

As with any modelling study, the approach presented here relies on various assumptions. For robust quantification of $R_{t}$ using renewal equations, reliable estimates of the generation-time distribution are required. Such estimates should account for the possibility that this quantity can change during an ongoing outbreak [31–34]. We additionally assumed that all infections are generated within the populations modelled; that is, there are no imported infections, and failing to account for their presence can lead to biases in inferred $R_{t}$ values [35]. An $R_{t}$ inference method based on our work here would also require estimates of the contact matrix and the relative infectiousness and susceptibility for those groups within the population under consideration; information on group-specific case counts would also provide useful information for $R_{t}$ quantification. We aim to explore the usefulness of such data in future work.

Additionally, the renewal equation model that we have derived is only suitable for modelling directly transmitted pathogens. Extending this work to model heterogeneities that are relevant for other pathogens, such as vector-borne diseases, is another possible target for future research. Another important limitation to using this general form of the renewal equation model is that we do not account for any differences in the rates of reporting of cases across the population groups—for example, for COVID-19, due to milder symptoms being expressed in children, infections in the younger population groups were reported less frequently than in the older adults. Not accounting for these differences could lead to larger bias in the $R_{t}$ estimation than by using the renewal equation model for a one-group population.

Despite these simplifications, our work provides a theoretical basis for future studies that account for heterogeneity in host populations when inferring $R_{t}$. Using our analytically derived expression for $R_{t}$, the effects of a range of interventions on the epidemic fate could be explored. Non-pharmaceutical interventions (NPIs) may impact population mixing patterns, which would change the effective contact matrix $C_{t}$ and, consequently, change $R_{t}$. This means that we can assess the impact of some NPIs on $R_{t}$ by simply changing the contact matrix structure. As an example, the impacts of interventions that affect particular age groups, such as school or workplace closures, could be explored by modifying the contact matrix [36]. Undertaking such an analysis in a realistic fashion would require real-time estimates of contact matrices, which could be provided from, for example, mobility data [37]. These types of analyses are also possible using next-generation matrix approaches [16]; by incorporating contact matrix structure information into a renewal equation framework, our approach is a hybrid between mechanistic, transmission dynamics models and more statistical approaches, such as the canonical, one-group renewal equation, which require fewer assumptions. This means the analyses it allows are complementary.

Renewal equation models have proved remarkable tools in epidemic surveillance, owing to the relatively few assumptions required to infer $R_{t}$. An implicit assumption underpinning the majority of their usage is, however, that there are no important heterogeneities in the population. Here, we show how to account for these heterogeneities in a renewal equation framework for structured populations. With the advent of real-time data on individual contact patterns, these modified renewal equation approaches could form the basis of future methods for inferring epidemiological quantities such as $R_{t}$. By enabling public health policy-makers to track transmission more accurately, such approaches have the potential to allow policy-makers to make the most informed possible decisions during future outbreaks of a wide range of pathogens.

## Data Availability

All data and computing scripts required to reproduce the results presented here are available on Zenodo [38].

## Footnotes

^1^
From now on we will refer to $C_{t}$ as the transposed of the contact matrix. In our analyses, in order to parametrize $C_{t}$ we will always use the transposed of the contact matrix from the POLYMOD study [11] if we use this type of data.

## Appendix A. Thieme’s renewal theorem

Here, we closely follow the more general approach presented in [39] for a continuous-time renewal equation model with multiple groups.

We reframe our modelling framework as follows:

$$\begin{array}{r} \left(\begin{array}{c} I^{1}(t)\\ \vdots \\ I^{N}(t) \end{array}\right)=\int_{a=0}^{\infty }K(a)\left(\begin{array}{c} I^{1}(t-a)\\ \vdots \\ I^{N}(t-a) \end{array}\right)\text{d}a, \end{array}$$

where the kernel of the renewal process is given by K(a)=γ(t)C(t)W(a). If the kernel satisfies a set of conditions (which we give below), then the long-term solution can be shown to follow:

$$\begin{array}{r} \left(\begin{array}{c} I_{t}^{1}\\ \vdots \\ I_{t}^{N} \end{array}\right)={\text{e}}^{rt}\underline{\Phi }. \end{array}$$

The kernel function must then satisfy the following five conditions:

(1) K(a) is a non-negative matrix for all a>0. This is true, since γ(t)>0, and W(a) and C(t) are non-negative matrices.
(2) An element of K(a) exceeds zero for some a>0. Similar to before, this is also satisfied as $\int_{a=0}^{\infty }K(a)\text{d}a=\gamma (t)C(t)\int_{a=0}^{\infty }W(a)\text{d}a=\gamma (t)C(t)$ has at least two non-zero elements (otherwise all contacts are 0).
(3) $\int_{a=0}^{\infty }∥K(a)∥da<\infty$, where we define the $L_{1}$-norm of a matrix M as $\|M||=\max_{1\leq i\leq N}\sum_{j=1}^{N}|M^{\left(ji\right)}|$ and $|M^{\left(ji\right)}|$ is the absolute value of the (j,i)th element of matrix M. This condition is also met: by replacing the kernel function evaluated at a with γ(t)C(t)W(a), the condition becomes
  $$\begin{array}{rl} \int_{a=0}^{\infty }∥K(a)∥\text{d}a & =\gamma (t)\int_{a=0}^{\infty }\max_{1\leq i\leq N}\sum_{j=1}^{N}\left|C^{\left(ji\right)}(t)w^{i}(a)\right|\text{d}a\\ & =\gamma_{t}(\int_{a=0}^{\infty }w^{i}(a)\text{d}a)\max_{1\leq i\leq N}\sum_{j=1}^{N}\left|C^{\left(ji\right)}(t)\right|\\ & =\gamma_{t}\max_{1\leq i\leq N}\sum_{j=1}^{N}C^{\left(ji\right)}(t)<\infty \end{array}$$
  as required. For the last two conditions, Boldin *et al.* [39] uses a discrete time renewal process. We follow with similar assumptions which we assume to hold in the continuous time case as well.
(4) There exists $a_{0}\in \mathbb{N}$ such that $R(a_{0})$ is a strictly positive matrix, where R(a)=R∗K(a)+K(a)=K∗R(a)+K(a),∀a>0 is defined as the resolvent of the kernel matrix K(a). We use R∗K(a) to mean $R*K(a)=\sum_{t=0}^{a}R(a-t)K(t)$ as described in [33]; also a strictly positive matrix implies that all its elements are greater than 0; that is $m_{ij}>0,\forall i,j$ for a strictly positive matrix $M=(m_{ij}{)}_{i,j}$.
(5) Additionally, there exists $a_{1}\in \mathbb{N}$ and $a_{2}\in \mathbb{N}$ with greatest common divisor $(a_{1},a_{2})=1$ such that both $R(a_{1})$ and $R(a_{2})$ have all elements greater than 0 and at least one element exceeding 0.

For the fifth condition, we recursively compute the values of resolvent matrix R(a). For a=0, we have that $R(0)=K(0)=O_{N}$ as seen in [39]. For the next value a=1, we have that R(1)=K(1)+W(0)R(1)+W(1)R(0)=K(1). As K(1)=γ(t)C(t)W(1) is a non-negative matrix, then R(1) is a non-negative matrix. From the definition of R(a), by induction we conclude that R(a) is a non-negative matrix for all a∈ℕ. We consider the first-time point $a^{\prime }$ for which $K(a^{\prime })$ has an element bigger than zero. From the second condition, we have that such an $a^{\prime }$ exists.

If $a^{\prime }=1$, we have that both $R(2)=K(2)+K(1)R(1)=K(2)+K(1{)}^{2}$ and $R(3)=K(3)+K(1)R(2)+K(2)R(1)=K(3)+K(1)K(2)+K(2)K(1)+K(1{)}^{3}$ will have at least one element greater than zero and are non-negative matrices, and as (2,3)=1*,* the desired fifth condition is satisfied.

If $a^{\prime }\geq 2$, however, $R(a^{\prime })=K(a^{\prime })+R*K(a^{\prime })$ has an element bigger than zero as $R*K(a^{\prime })$ is itself a non-negative matrix. Additionally, $R(a^{\prime }+1)=K(a^{\prime }+1)+K(a^{\prime })R(1)+\ldots$ $K(1)R(a^{\prime })=K(a^{\prime }+1)+K(a^{\prime })K(1)=K(a^{\prime }+1)$ as the condition on the value of $a^{\prime }$ implies that K(a) is the identical zero matrix $\forall a<a^{\prime }$ and R(1)=K(1).

In terms of the fourth condition, we saw from before that R(a) can be written as a sum of powers of the kernel matrices K(b) for some bs with b≤a for all a∈ℕ. The kernel matrices K(a)=γ(t)C(t)W(a), where W(a) is a diagonal matrix. If all population groups mix between each other, the contact matrix C(t) will be strictly positive. Therefore, $K(a^{\prime })$ will also be strictly positive, satisfying the fourth condition.

Otherwise, if the modelled population includes demographic groups that do not interact with any other groups, then the contact matrix C(t) is a diagonal matrix and can be written as

(A 1)
$$C(t)=\left(\begin{array}{cccc} c_{11} & & & \\ & \ddots & & 0\\ & & c_{mm} & \\ & 0 & & C^{*}(t) \end{array}\right).$$

As W(a) is a diagonal matrix itself, then we would have that the kernel matrix evaluated for any time a is equal to

$$K(a)=\gamma (t)\left(\begin{array}{cccc} c_{11}w^{1}(a) & & & \\ & \ddots & & 0\\ & & c_{mm}w^{m}(a) & \\ & 0 & & C^{*}(t)W_{m:,m:}(a) \end{array}\right).$$

Regardless of how many times this type of matrix is raised to any power and how many times we sum it to compute R(a), the top block of the resulting matrix will remain a diagonal matrix and therefore not satisfying the fourth condition imposed. Hence, our findings are confined to only fully mixing populations.

For example, suppose that the total contact matrix, C(t), is diagonal, meaning that the groups do not interact. In this case, it is not generally true that the growth rate of new infections should be identical across groups, as equation (3.10) supposes. For example, if groups have differing generation times and they do not interact, the infections will generally grow at different calendar rates in each group.

## Appendix B. Renewal equations and reproduction numbers for a discrete-time structured population model

When inferring $R_{t}$, the renewal equation frameworks typically used are discrete in time (usually with time steps of 1 day; see §2). We now demonstrate that for a discrete-time version of the model presented so far, we obtain an analogous renewal equation as for the continuous-time model and the same expression for $R_{t}$.

When time and age are discrete, the analogous conservation equation for the population is a difference equation:

$$\begin{array}{r} \frac{n(t+\Delta t,a+\Delta a)-n(t,a+\Delta a)}{\Delta t}+\frac{n(t,a+\Delta a)-n(t,a)}{\Delta a}=0, \end{array}$$

where Δt=Δa represents a discrete unit of time. The density of new infections occurring at a time t=TΔt is

(B 1)
$$\begin{array}{lcr} & n(T\Delta t,0)=\sum_{A=0}^{\infty }C_{t}\gamma_{t}w(A\Delta t)n(T\Delta t,A\Delta t). & \end{array}$$

Similar to the continuous-time population model, we only consider infections occurring after time t=TΔt=0. The initial number of infections at time T=0 can be written as n(0,AΔt)=f(AΔt). The analogous characteristics for this discrete-time model are

(B 2)
$$a=A\Delta t=\left\{ \begin{array}{l} T\Delta t+A_{0}\Delta t,\text{ for }a>t,\text{ i.e.\textbackslash{} }A>T\\ T\Delta t-T_{0}\Delta t,\text{ for }a\leq t,\text{ i.e.\textbackslash{} }A\leq T, \end{array}\right.$$

where $A_{0}>0$ is the number of age units of an infection at time t=0 and $T_{0}\geq 0$ is the number of units of time at which an infection occurred. This allows us to define the number of infections as a function of t only, that is we can write n(TΔt,AΔt)=n(TΔt,A(T)Δt):=n(TΔt)*,* for a given choice of $A_{0}$, or $T_{0}$ respectively. Along each such defined *characteristic* lines, the number of infections is determined by a singular point in time τ, with $\tau =A_{0}\Delta t$ uniquely for A>T and $\tau =T_{0}\Delta t$ for A≤T. Therefore, the number of infections is conserved along each characteristic line, i.e.

$$\begin{array}{c} n(t,a(t))=\text{const.} \end{array}$$

Using equation (B 2), we derive the constant population sizes: when A>T, A can be written $a=A\Delta t=T\Delta t+A_{0}\Delta t$ which implies $n(T\Delta t,T\Delta t+A_{0}\Delta t)=\text{const.}$; similarly when T=0, the population size is

(B 3)
$$\begin{array}{lcr} & n(0,A_{0}\Delta t)=f(A_{0}\Delta t)=f((A-T)\Delta t). & \end{array}$$

When A≤T, we use instead the fact that $A\Delta t=T\Delta t-T_{0}\Delta t$; as $n(t,t-T_{0}\Delta t)=\text{const.}$*,* and, when $T=T_{0}$:

(B 4)
$$\begin{array}{lcr} & n(T_{0}\Delta t,0)=n((T-A)\Delta t,0). & \end{array}$$

Collecting equations (B 3) and (B 4), the solutions for the discrete population sizes for all infection onset times are given by either of the following two forms:

(B 5)
$$\begin{array}{lcr} & n(T\Delta t,A\Delta t)=\left\{ \begin{array}{c} f((A-T)\Delta t),A>T,\\ n((T-A)\Delta t,0),A\leq T. \end{array}\right. & \end{array}$$

Substituting the equation (B 5) back into (B 1), we obtain that

$$\begin{array}{rl} n(T\Delta t,0) & =\sum_{A=0}^{T}b(A\Delta t)n(T\Delta t,A\Delta t)+\sum_{A=T+1}^{\infty }b(A\Delta t)n(T\Delta t,A\Delta t)\\ & =\sum_{A=0}^{T}b(A\Delta t)n((T-A)\Delta t,0)+\sum_{A=T+1}^{\infty }b(A\Delta t)f((A-T)\Delta t). \end{array}$$

Since similarly to the continuous-time case, for any $A^{\prime }>T$, b(AΔt)≈0, the number of new infections observed at time t=TΔt becomes

$$\begin{array}{rl} n(T\Delta t,0) & =\sum_{A=0}^{T}b(A\Delta t)n((T-A)\Delta t,0)+\sum_{A=T+1}^{\infty }0f((A-T)\Delta t)\\ & =\sum_{a=0}^{t}\gamma_{t}C_{t}w_{a}n(t-a,0)=\sum_{a=0}^{t}\gamma_{t}C_{t}w_{a}I_{t-a}, \end{array}$$

which is indeed identical to the deterministic form of the renewal equation process as described in equation (2.1). The rest of the proof for the definition of the reproduction number and epidemic stability criterion $R_{t}=\sum_{a=0}^{\infty }\gamma_{t}C_{t}w_{a}=\gamma_{t}C_{t}\sum_{a=0}^{\infty }w_{a}=\gamma_{t}C_{t}$ follows according to the methodology presented in [39].

For the multiple-group population, a similar argument emerges [39]—we arrive at the conclusion that the overall reproduction number $R_{t}=\rho (\sum_{a=0}^{\infty }\gamma_{t}C_{t}W_{a})=\rho (\gamma_{t}C_{t}\sum_{a=0}^{\infty }W_{a})=\rho (\gamma_{t}C_{t})=\gamma_{t}\rho (C_{t})$ is a correct criterion for the long-term behaviour of an epidemic.

## Appendix C. The typical generation time interval affects epidemic growth rates in calendar time

In electronic supplementary material, figure S1, we simulate the trajectories of the total number of new infections for a range of values of the generation time interval means:

$${\bar{W}}_{1}=\left(\begin{array}{c} 3.3\\ 5\\ 7 \end{array}\right),\;{\bar{W}}_{2}=\left(\begin{array}{c} 15.3\\ 15.3\\ 15.3 \end{array}\right),\;\text{and}\;{\bar{W}}_{3}=\left(\begin{array}{c} 30\\ 25.3\\ 15 \end{array}\right).$$

In each case, the standard deviation of the generation time distribution was assumed to be 3.3 days.

We use the contact matrix given by equation (3.11). The infection rate γ(t) is also kept constant for all plotted scenarios. In the beginning of the simulation, we see the different oscillatory patterns for each of our choices of the generation time intervals. However, as the time increases, for all R(t) values and all selected Ws, the oscillations die out and the number of new infections approach 0 (panel A; epidemic decay), reach stable values (panel B; epidemic persistence) or continue to increase exponentially at a constant rate (panel C; epidemic growth).

## Appendix D. $R_{t}$ for a discrete-time stochastic model

We now derive $R_{t}$ for a stochastic version of a renewal model. We consider only a renewal equation for a population comprising members of a single group since these results directly translate to the multiple-group case. The renewal equation we assume is of the form

(D 1)
$$\begin{array}{lcr} & I_{t}∼\text{Poisson}\left(R\sum_{s=1}^{p}\omega_{s}I_{t-s}\right). & \end{array}$$

This means that for an arbitrary time t+τ, we have

(D 2)
$$\begin{array}{lcr} & I_{t+\tau }∼\text{Poisson}\left(R\sum_{s=1}^{p}\omega_{s}I_{t+\tau -s}\right). & \end{array}$$

Taking expectations at time t yields

(D 3)
$$\begin{array}{lcr} & E_{t}\left[I_{t+\tau }\right]=R\sum_{s=1}^{p}\omega_{s}E_{t}\left[I_{t+\tau -s}\right]. & \end{array}$$

Then, denoting $M_{j}:=E_{t}\left[I_{t+\tau }\right]$ as the expectation at time t of the infection count at time t+τ yields a renewal equation in terms of this quantity:

(D 4)
$$\begin{array}{lcr} & M_{j}=R\sum_{s=1}^{p}\omega_{s}M_{j-s}. & \end{array}$$

We then suppose a long-run solution of the form $M_{j}\approx \lambda^{j}$, which results in

(D 5)
$$\begin{array}{lcr} & \lambda^{j}=R\sum_{s=1}^{p}\omega_{s}\lambda^{j-s}\Rightarrow 1=R\sum_{s=1}^{p}\omega_{s}\lambda^{-s}. & \end{array}$$

We can then define

(D 6)
$$\begin{array}{lcr} & K(\lambda )=R\sum_{s=1}^{p}\omega_{s}\lambda^{-s}. & \end{array}$$

By construction, K(1)=R and K(λ) is a decreasing function of λ. This means that

— If R>1, $\lambda^{*}=\lambda \text{ s.t. }K(\lambda )=1$ must satisfy $\lambda^{*}>1$,
— If R≤1, $\lambda^{*}\leq 1$,

and similarly so for complex λ (see [39, §2.1]). This means that we can consider R to satisfy the threshold conditions governing the long-run fate of the stochastic mean in the stochastic renewal model.

## Appendix E. Doubling times

We derive the relationship between the reproduction number and the doubling time $\tau_{0}:=\frac{1}{r}\ln(2)$—the time taken for an epidemic to double in size. Rearranging this expression and substituting it in place of r in equation (3.19) results in

$$\begin{array}{r} \frac{1}{R(t)}=\frac{\rho (C(t)\int_{0}^{\infty }W(a)2^{-\tau_{0}^{-1}a}\text{d}a)}{\rho (C(t))}. \end{array}$$

## Appendix F. The relationship between R(t) and the growth rate for the discrete-time deterministic model

We derive the relationship between R(t) and the growth rate r for structured populations in the case of the discrete-time deterministic model. From [39] §2.2 and appendix B the overall reproduction number of the system is defined by R(t)=ρ(K(1)), where K(λ) is defined as

$$\begin{array}{r} K(\lambda )=\gamma (t)C(t)\sum_{a=0}^{\infty }W(a)\lambda^{-a}; \end{array}$$

this definition for K(λ) is simply the multivariate equivalent of equation (D 6). Similar to appendix D, λ is defined in terms of the long-term solution of the infection vector count, that is $\underline{I}{\;}_{j}=\lambda^{j}\underline{\Phi }$, where $\underline{\Phi }$ is a constant non-negative vector for large values of j. For the discrete-time deterministic model, the overall growth rate r is defined such that $\underline{I}{\;}_{t+1}=(1+r)\underline{I}{\;}_{t}$, therefore implying that λ=1+r. Hence, we have the following system of identities:

(F 1)1=ρ(K(λ))=γ(t)ρ(C(t)∑a=0∞W(a)λ−a)=γ(t)ρ(C(t)∑a=0∞W(a)(1+r)−a)(F 2)Rt=ρ(K(1))=γ(t)ρ(C(t)∑a=0∞W(a))=γ(t)ρ(C(t)),

which when divided produce equation (3.20).

## Appendix G. Empirical estimates of the growth rate from simulations of the stochastic model

In order to determine the empirical growth rate r of a population for which we know its overall reproduction number R(t), we use the *scipy.integrate* and *scipy.optimize.minimize* functions [40]. The goal function for the optimizer is given by the following equation in terms of r derived from equation (3.19):

$$\begin{array}{r} f(r):=\left|\frac{\rho (C(t))}{\rho (C(t)\int_{0}^{\infty }W(a)\exp(-ra)\text{d}a)}-R(t)\right|. \end{array}$$

The optimizer function will then return the value of r for which f(r) is closest to zero and thus approximates the empirical growth rate of the population thus simulated.

## Appendix H. Identical rows matrix spectral radius

We are computing the spectral radius of a positive matrix with identical rows, which is defined as the maximum real eigenvalue of the matrix. That is, the maximum value of λ where

$$\begin{array}{r} \left[\begin{array}{ccc} a_{1} & \ldots & a_{N}\\ \vdots & \ldots & \vdots \\ a_{1} & \ldots & a_{N} \end{array}\right]\left(\begin{array}{c} x_{1}\\ \vdots \\ x_{N} \end{array}\right)=\left(\begin{array}{c} a_{1}x_{1}+\ldots a_{N}x_{N}\\ \ldots \\ a_{1}x_{1}+\ldots a_{N}x_{N} \end{array}\right)=\lambda \left(\begin{array}{c} x_{1}\\ \vdots \\ x_{N} \end{array}\right) \end{array}$$

which implies $a_{1}x_{1}+\ldots a_{N}x_{N}=\lambda x_{i},\forall i$. One solution is for λ=0, which implies that $a_{1}x_{1}+\ldots a_{N}x_{N}=0$. Otherwise, if λ≠0 then that implies that $x_{1}=\ldots x_{N}$, therefore $a_{1}x_{1}+\ldots a_{N}x_{N}=(\sum_{i=1}^{N}a_{i})x_{1}=\lambda x_{1}$. Since we cannot have $x_{1}=0$ (otherwise the eigenvector is the zero-vector, contradiction), then the only other eigenvalue of the matrix is given by $\lambda =\sum_{i=1}^{N}a_{i}>0$ by the definition of the positive matrix. Therefore, the spectral radius of the matrix

$$\rho (\left[\begin{array}{ccc} a_{1} & \ldots & a_{N}\\ \vdots \; & \ldots & \vdots \;\\ a_{1} & \ldots & a_{N} \end{array}\right])=\sum_{i=1}^{N}a_{i}.$$

## Appendix I. Exact overall reproduction number for a two-group population

For the more general behaviour of a two group population, we use the property that the spectral radius of a general 2 × 2 matrix

$$M=\left[\begin{array}{cc} a & b\\ c & d \end{array}\right]$$

is given by the maximum eigenvalue, i.e. $\max(\frac{a+d\pm \sqrt{(a-d{)}^{2}+4bc}}{2})=\frac{a+d+\sqrt{(a-d{)}^{2}+4bc}}{2}$. Therefore, if the contact matrix of the population is given by

$$C(t)=\left[\begin{array}{cc} c_{11} & c_{12}\\ c_{21} & c_{22} \end{array}\right]$$

then we have that $\rho (C(t))=\frac{c_{11}+c_{22}+\sqrt{(c_{11}-c_{22}{)}^{2}+4c_{12}c_{21}}}{2}$ and similarly, using the fact that

$$C(t)\int_{0}^{\infty }w(a){\text{e}}^{-ra}\text{d}a=\left[\begin{array}{cc} c_{11}\int_{0}^{\infty }w^{1}(s){\text{e}}^{-ra}\text{d}a & c_{12}\int_{0}^{\infty }w^{2}(s){\text{e}}^{-ra}\text{d}a\\ c_{21}\int_{0}^{\infty }w^{1}(s){\text{e}}^{-ra}\text{d}a & c_{22}\int_{0}^{\infty }w^{2}(s){\text{e}}^{-ra}\text{d}a \end{array}\right]$$

implies that

$$\rho \left(C(t)\int_{0}^{\infty }w(a){\text{e}}^{-ra}\text{d}a\right)=\frac{\sqrt{\begin{array}{l} \;\;{\left(\int_{0}^{\infty }\left(c_{11}w^{1}(s)-c_{22}w^{2}(s)\right){\text{e}}^{-ra}\text{d}a\right)}^{2}\\ +4\left(c_{12}\int_{0}^{\infty }w^{1}(s){\text{e}}^{-ra}\text{d}a\right)\left(c_{21}\int_{0}^{\infty }w^{2}(s){\text{e}}^{-ra}\text{d}a\right)\\ \;\;\;+\int_{0}^{\infty }\left(c_{11}w^{1}(s)+c_{22}w^{2}(s)\right){\text{e}}^{-ra}\text{d}a \end{array}}}{2}.$$

Therefore, the relationship between the overall reproduction number γ(t) and epidemic growth rate r is given by

$$\frac{1}{R(t)}=\frac{\sqrt{\begin{array}{l} \;\;{\left(\int_{0}^{\infty }\left(c_{11}w^{1}(s)-c_{22}w^{2}(s)\right){\text{e}}^{-ra}\text{d}a\right)}^{2}\\ +4\left(c_{12}\int_{0}^{\infty }w^{1}(s){\text{e}}^{-ra}\text{d}a\right)\left(c_{21}\int_{0}^{\infty }w^{2}(s){\text{e}}^{-ra}\text{d}a\right)\\ \;\;\;+\int_{0}^{\infty }\left(c_{11}w^{1}(s)+c_{22}w^{2}(s)\right){\text{e}}^{-ra}\text{d}a \end{array}}}{c_{11}+c_{22}+\sqrt{{\left(c_{11}-c_{22}\right)}^{2}+4c_{12}c_{21}}}.$$

More particularly if

$$C_{t}=c\left[\begin{array}{cc} 2 & 1\\ 1 & 1 \end{array}\right]$$

then this relationship becomes

$$\begin{array}{rl} \frac{1}{R(t)} & =\frac{\int_{0}^{\infty }(2cw^{1}(s)+cw^{2}(s)){\text{e}}^{-ra}\text{d}a+\sqrt{4c^{2}(\int_{0}^{\infty }w^{1}(s){\text{e}}^{-ra}\text{d}a{)}^{2}+c^{2}(\int_{0}^{\infty }w^{2}(s){\text{e}}^{-ra}\text{d}a{)}^{2}}}{3c+c\sqrt{5}}\\ & =\frac{\int_{0}^{\infty }(2w^{1}(s)+w^{2}(s)){\text{e}}^{-ra}\text{d}a+\sqrt{4(\int_{0}^{\infty }w^{1}(s){\text{e}}^{-ra}\text{d}a{)}^{2}+(\int_{0}^{\infty }w^{2}(s){\text{e}}^{-ra}\text{d}a{)}^{2}}}{3+\sqrt{5}}. \end{array}$$

## Appendix J. Varying generation time distributions across groups

We now explore how the relationship between r and R(t) is affected by the composition of generation time distributions across the groups.

We consider three scenarios making different assumptions about the generation time distributions in each age group. For each scenario and for each group, we assume the generation time distribution is governed by a (discretized) gamma distribution with the same standard deviation, of 3.3 days; the mean generation time is allowed to vary by group. In the first scenario, we assume the mean generation time is lowest in the youngest age group and highest in the eldest; in the second, the mean generation time is the same in each group; in the third scenario, the generation time decreases with age. The means in each scenario are given in table 1.

In electronic supplementary material, figure S2, we show the relationship between epidemic growth rate r and R(t) implied by equation (3.19) according to each of the three scenarios. In all cases, r=0 implies R(t)=1. Since the 0−20 age group has the highest contact rates, most infections are generated from this group and it has the single greatest contribution to R(t). Because of this, changes in the generation time of this group skew the relationship between r and R(t): when the generation time interval is short in this group, the corresponding growth rate is high; and this decreases as their generation time is increased.

To further highlight this sensitivity, in electronic supplementarty material, figure S3, we show how the epidemic growth rate responds to changes in the mean generation time in those aged 0−20 (left-hand panel) and those aged 65+ (right-hand panel); in each case, the mean generation time in the other groups remains fixed at 15.3 days. We consider three values of R(t) for each panel (corresponding to the coloured lines).
